# Factors Associated with Adherence to Treatment in Patients with HIV and Diabetes Mellitus

**DOI:** 10.3390/jpm13020269

**Published:** 2023-01-31

**Authors:** Cristina Rivera-Picón, María Hinojal Benavente-Cuesta, María Paz Quevedo-Aguado, Juan Luis Sánchez-González, Pedro Manuel Rodríguez-Muñoz

**Affiliations:** 1 Faculty of Health Sciences, Nursing, Pontifical University of Salamanca, 37002 Salamanca, Spain; 2 Faculty of Nursing and Physiotherapy, University of Salamanca, 37008 Salamanca, Spain; 3 Department of Nursing, Instituto Maimónides de Investigación Biomédica de Córboda, 14005 Córdoba, Spain

**Keywords:** nurses, nursing, adherence to treatment, chronic diseases, HIV, diabetes mellitus

## Abstract

We aim to identify the factors that influence the therapeutic adherence of subjects with chronic disease. The design followed in this work was empirical, not experimental, and cross-sectional with a correlational objective. The sample consisted of a total of 400 subjects (199 patients with HIV and 201 patients with diabetes mellitus). The instruments applied for data collection were a sociodemographic data questionnaire, the 4-item Morisky Medication Adherence Scale (MMAS-4) and the Coping Strategies Questionnaire. In the group of subjects with HIV, that the use of emotional coping strategies was related to lower adherence to treatment. On the other hand, in the group of subjects with diabetes mellitus, the variable related to compliance with treatment was the duration of illness. Therefore, the predictive factors of adherence to treatment were different in each chronic pathology. In the group of subjects with diabetes mellitus, this variable was related to the duration of the disease. In the group of subjects with HIV, the type of coping strategy used predicted adherence to treatment. As a result of these results, it is possible to develop health programmes to promote, from nursing consultations to adherence to treatment of patients with HIV and diabetes mellitus.

## 1. Introduction

Currently, we are facing an increase in life expectancy and a clear ageing of the world’s population. This has led to a significant increase in the incidence of chronic diseases, which represent a public health problem [1]. In this way, subjects with chronic pathologies must have good management of their disease to minimize its impact, improve health outcomes, prevent further disability and reduce healthcare costs. In this context of study, the concept of therapeutic regimen management is presented as a key component in the management of these diseases [2]. However, a high number of subjects with chronic pathologies do not comply with treatment adequately, and the rate of adherence to it in this type of patient is low [3]. 

Based on these assumptions, this study wishes to determine the factors that are related to better drug adherence in certain chronic diseases. In addition, knowing these factors is essential to achieve adequate management of the therapeutic regimen. This is justified because, in order to favor the management of the therapeutic regimen, it must be taken into account that health must be centered on the patient, for which reason their active collaboration is needed, taking into account their fears, their will or their difficulties in the treatment [4]. 

However, it should be noted that each chronic pathology has clinical characteristics and a particular therapeutic plan, which is why we cannot include all of them under the same heading of “chronicity”. Consequently, in this study we have considered it necessary to specifically analyse adherence to treatment in two specific chronic diseases: HIV and diabetes mellitus.

HIV remains a relevant chronic disease worldwide. This infection, has had an enormous impact on morbidity, the demography and economy of the most affected countries. The latest data recorded in Spain show that 3244 new cases of HIV were diagnosed since 2019 [5].

The introduction of highly active antiretroviral therapy (HAART) has modified the natural history of HIV infection, despite the adverse reactions associated with such treatment [6,7]. The antiretroviral treatments currently used allow for reducing transmission rates, as well as increasing the survival of patients diagnosed with HIV [8]. In this way, this infection has become a chronic disease [9]. There is evidence that the incorrect taking of antiretroviral treatment is associated with the appearance of viral strains resistant to it, increasing the risk of disease progression. Therefore, despite the clear advantages of the treatment, it is essential to maintain correct management of the therapeutic regimen [10,11,12]. However, most research on subjects with HIV indicates that adherence to treatment in this group of patients is low [9,13,14].

On the other hand, diabetes mellitus is one of the most prevalent chronic diseases diagnosed today. In 2002, the WHO announced a global prevalence of diabetes of 3%, which corresponds to 170 million people in the world diagnosed with this pathology [15]. It was even estimated that this figure would more than double by the year 2025 [16]. Currently, these forecasts have already been exceeded, since the latest figures provided by the International Diabetes Federation (IDF), corresponding to 2019, state that 9.3% of adults between 20 and 79 years old have diabetes, which corresponds to a total of 463 million people. Based on this data, the IDF estimates that 578 million adults will live with the disease in 2030. In 2045, they estimate that the figure will rise to 700 million [17]. Through the National Health Survey/European Health Survey in Spain (ENS/EESN), estimates of the prevalence of diabetes in Spain have also been obtained. The last one, corresponds to the year 2017, and states that the prevalence has almost doubled in Spain between 1993 (4.1%) and 2017 (7.8%). In subjects with diabetes, the importance of adherence to treatment to achieve adequate metabolic control is highlighted, which allows for delaying and preventing the development of complications associated with this disease [18,19]. However, it has been described that pharmacological compliance in diabetic subjects is also low [20,21].

Therefore, there is no doubt that incorrect adherence to treatment is a major health problem. Thus, the work of professionals is important to guarantee individualized and comprehensive patient care, allowing their active participation and improving the therapeutic relationship [22]. In addition, both in diabetic subjects and in those with a diagnosis of HIV, the adherence to treatment is a highly complex phenomenon, in which different variables may intervene [23]. Among these, factors related to the treatment itself stand out, such as the duration of illness, number of doses or associated side effects [24]. On the other hand, psychosocial factors are reflected, among which coping strategies have stood out. Certain coping strategies have been described as possible health protective factors, indicating their relationship with adherence to pharmacological treatment. Thus, it is stated that low levels of adherence to treatment are related to the use of emotional or avoidant strategies, while rational coping strategies can predict high adherence to treatment in certain chronic pathologies [25,26]. On the other hand, in relation to the sociodemographic variables, there was more heterogeneity of evidence, finding different results on the relationship between sex, marital status, educational level and the age of the patient with the level of adherence to treatment [27,28,29].

Consequently, the objective of this study was to identify those factors associated with adherence to drug treatment in subjects with diabetes mellitus or HIV. This could guide health teams in planning strategies that favor the management of the therapeutic regimen, through a more active collaboration of the patient.

## 2. Materials and Methods

### 2.1. Design 

The study had a non-experimental cross-sectional design, with a correlational objective.

### 2.2. Participants 

Subjects diagnosed with Diabetes or HIV+. The sample was collected at the University Hospital of Salamanca (HUS) and at different Primary Care centers in that province.

In the first place, the sample of subjects with HIV was obtained through an incidental sampling, in the infectious diseases unit of the Salamanca clinical hospital. Subsequently, we obtained the sample of subjects with diabetes. A quota sampling was applied in order to obtain homogeneous subsamples. Said subsample of diabetic subjects was defined as having a size equivalent to that of subjects with HIV. The collection of the subsample of diabetic patients was carried out from different Internal Medicine floors of the Clinical Hospital of Salamanca and different Salamanca health centres (namely, “Periurbana Sur” and “Capuchinos” Health Centres). 

The following inclusion criteria were having a confirmed diagnosis of the disease (HIV or diabetes), regardless of its stage. They also had to be in current treatment, be of legal age and voluntarily participate in the study. As exclusion criteria, any illness or disorder that would prevent the patient from signing the informed consent was considered or completing the study.

### 2.3. Data Collection 

The data was collected taking a total of two years to collect them (2018–2020). The questionnaires used were:

#### 2.3.1. Sociodemographic Data Questionnaire

The variables collected through this instrument were of a sociodemographic nature and information related to health:

Type of disease (HIV or diabetes)

Duration of diagnosis of the disease (1–5 years, 5–10 years or more than 10 years)

Sociodemographic variables studied (age, sex, marital status and educational level)

#### 2.3.2. 4-Item Morisky Medication Adherence Scale (Morisky-Green-Levine)

The questionnaire originally developed by Morisky et al. (1986) under the name “4-item Morisky Medication Adherence Scale” (MMAS-4). In Spanish, this questionnaire was validated by Val-Jiménez et al. (1992). The scale is made up of four questions with a dichotomous response format (Yes/No), which reflects the patient’s behaviour regarding compliance. The patient shows good adherence to treatment when he/she correctly answers the four questions (No/Yes/No/No), corresponding to poor adherence if he/she answers three or fewer questions adequately. Therefore, through this questionnaire, patients are categorized as adherent to treatment or nonadherent to treatment [30].

Originally, the Morisky-Green-Levine treatment adherence questionnaire had adequate psychometric properties with high discrimination ($\rho_{bp}$ = 0.43), high sensitivity (sensitivity = 0.81) and low specificity (specificity = 0.44). In Spain, this test was validated by Val-Jiménez et al. (1992) with a sample of hypertensive patients. The sensitivity was somewhat lower, while the specificity value was practically the same (sensitivity = 0.52, specificity = 0.44).

#### 2.3.3. Coping Strategies Questionnaire (Sandín and Chorot)

Coping styles were measured by means of the structured self-assessment known as the Coping Strategies Questionnaire (Cuestionario de Afrontamiento del Estrés (CAE)). The scale assesses seven coping factors: social support seeking, religious coping, overt emotional expression, avoidance coping, problem-solving coping, positive reappraisal and negative auto-focused coping. Also, it allows measuring the two most general dimensions, evaluated in our study: Rational Coping and Emotional Coping. The scale is made up of 42 items with a response range that goes from 0, never, to 4, almost always The total variance explained by the two general dimensions (Rational and Emotional) was 49.3% [31].

### 2.4. Ethical Considerations

This study obtained a favourable report from the Ethics Committee “Research with Medicines in the Salamanca Health Area” (CEIC code: PI02/01/2018). In addition, it received authorization by the Healthcare Complex of the University of Salamanca and by the Salamanca Primary Care Directorate. 

### 2.5. Data Analysis 

Data analysis was performed using the Statistics Package for the Social Sciences (SPSS) version 25 (IBM Corp, Armonk, NY, USA).

A descriptive analysis of the sociodemographic variables was carried out, analyzing the differences between the subsamples. For this, Pearson’s $\chi^{2}\;$ test was applied, determining the size of the effect through Cramer’s V.

To identify the factors that influence or help predict therapeutic adherence in subjects diagnosed with HIV and diabetes mellitus, logistic regression analysis was used. In this case, for each group of subjects with diabetes and HIV, a logistic regression analysis was carried out. The dependent variable was adherence to the treatment on the part of patients. This variable had two values, presence or absence of adherence. The independent variables were the type of coping strategy, sex, educational level, age, duration of illness and marital status.

The level of statistical significance used throughout the study was 0.05, with a 95% confidence interval.

## 3. Results

### 3.1. Descriptive Analysis

#### 3.1.1. Variables Related to the Type of Disease

A total of 400 patients participated in the present study. Depending on the type of disease of the subjects, two groups are differentiated: HIV patients (N = 199) and diabetics (N = 201). Marital status was the only significant variable ($\chi^{2\;}$= 42.484; *p* < 0.01). However, Cramer’s V value (V = 0.322) reflects that the effect is moderate. Most of the participants are male (N = 294), with mainly secondary or lower education (N = 328) and with a mean age of between 44 and 50 years (N = 124). Also, we observe that separated or divorced subjects are a minority (N = 38) (Table 1).

#### 3.1.2. Variables Related to Adherence to Treatment

The final sample consisted of 400 subjects, with 66% showing nonadherent behaviour. Table 2 shows the descriptions of the sociodemographic variables based on adherence. No significant differences were found in any of the sociodemographic variables regarding adherence to treatment.

### 3.2. Factors Predicting Treatment Adherence

#### 3.2.1. Subjects with HIV

Table 3 shows the values of the covariates before becoming part of the model. The only variables that are related to adherence to treatment in subjects with HIV were emotional coping and rational coping (*p* < 0.05).

The regression by specific steps showed that the best model was the one that only included the covariate use of emotional coping strategies (B = −0.065, ET = 0.024, Wald = 7.501, *p* = 0.006). Table 4 shows the values of the final model. It can be observed that the greater the use of emotional coping strategies in patients with HIV was, the lower the adherence to treatment (B = −0.065, Exp(B) = 0.937). 

Table 5 shows the results of different tests to assess the adequacy of the model. With a cut-off point of 0.6, about 66% of patients were correctly classified as adherent or nonadherent. Compared with the null model, the mismatch was reduced by 5.3% when the emotional coping variable was included. Finally, the Hosmer-Lemeshow test was not significant (*p* = 0.789). All these results led to the idea of a good fit of the model.

#### 3.2.2. Subjects with Diabetes

Table 6 shows the independent variable values in the null model. The only variable that improved the prediction of the null model was the duration of disease (*p* < 0.05).

Table 7 shows the results of the independent variables that have intervened in the equation. The only variable that was significant was the duration of disease. More specifically, the proportion of patients who were adherent was the same between people with 1–5 years of disease and 5–10 years of disease (*p* > 0.05). However, the proportion of patients who were adherent was higher among patients with more than 10 years of disease duration compared to that among patients with 1–5 years of disease duration (B = 0.275, ET = 0.358, Wald = 0.590, *p* = 0.443). The odds of showing adherence with more than 10 years of disease duration was 2.65 times the odds of adherence with 1–5 years of disease duration.

Table 8 shows the results of different tests to assess the adequacy of the model. When interpreting the goodness of fit through Nagelkerke’s R2, we observed that the mismatch was reduced by 4.8%. Furthermore, 60.5% of well-predicted cases were detected in the alternative model, using a 0.6 cut-off point for classification.

## 4. Discussion

In the present study, it was observed that a greater use of emotional coping strategies was related to lower adherence to treatment among subjects diagnosed with HIV. However, this variable was not relevant to determine adherence to treatment in subjects with diabetes. On the other hand, the duration of disease was related to the presence of adherence to treatment in subjects with diabetes, observing that adherence was more likely among patients with more than 10 years of disease duration than among patients with 1–5 years of disease duration. These results were not found in the sample of patients with HIV.

In addition, the sociodemographic variables studied, such as sex, marital status, age or educational level of the subjects, did not show a significant relationship with compliance with pharmacological treatment in either of the two subsamples.

In comparison with other studies, as we have indicated in the background of the article, some authors have pointed out the relationship between active or rational coping strategies with high adherence to treatment. In a complementary manner, others highlight that low levels of adherence are related to avoidant and emotional coping strategies, which are also called passive, palliative or maladaptive Strategies [32]. We highlight that these data have been confirmed in different investigations with certain chronic pathologies, such as kidney disease, multiple sclerosis or HIV [25,26,33].

In relation to the pathologies addressed in our study, we highlight the research of Weaver et al. (2005) carried out on subjects with HIV, which showed that coping strategies aimed at problem-solving were not related to adherence to HAART [34]. These results are similar to those found in our research, where only emotional coping strategies predicted the level of adherence. However, Delmas et al. (2008) did detect that those subjects with HIV who used active coping strategies experienced higher levels of adherence to treatment. It should be noted that, although the coping variable focused on the solution of the problem was significant considered in isolation in our results, it was not included in the final model [35].

Different investigations carried out in subjects with HIV have confirmed that the use of emotional, passive or maladaptive coping strategies are associated with a lack of adherence to treatment [36,37,38]. These results are consistent with those obtained in our research.

In parallel, the results identified in various projects involving patients with diabetes also coincided with those obtained in our study. In this way, different authors defend that there is no statistically significant relationship between the use of different types of coping strategies and adherence [39,40,41,42].

In relation to sociodemographic variables, there is more heterogeneity of results, both in subjects with diabetes mellitus and in those with a diagnosis of HIV.

Based on age, there is no consensus as to its role in adherence, since some studies confirm that adherence increases as age increases [29,43,44] and other investigations affirm the opposite [45].

The level of studies has also been proposed as a factor related to adherence in chronic diseases, highlighting less adherence at a lower level of studies [45,46,47]. However, other investigations present opposite data, justifying that subjects with a high level of education present with multiple responsibilities and occupations that require a lot of time and attention, which negatively interferes with their adherence [48]. Other research with diabetic subjects indicates that the titration does not predict adherence to treatment [44]. The study by Quiñones et al. (2018) only found a relationship between said variable and adherence in those diabetic patients who also suffered kidney damage [49]. Similarly, we have found studies on patients with HIV that also indicated the level of education is not related to adherence [29].

Studies that relate gender and adherence also do not offer conclusive results on the relationship between both variables. Thus, while some projects defend that it is women who have the greatest adherence [44], others affirm the opposite [50].

Based on marital status, we found authors who observe that married or partnered subjects have better adherence to treatment [51]. However, many others defend that it is not a variable associated with the adherence of patients with chronic diseases [52,53].

By way of summary, there are studies that affirm that sociodemographic variables are not related to the level of adherence to treatment. These results are consistent with those of our study. Thus, investigations carried out with samples of subjects with HIV found that age, sex, marital status, educational level and treatment time did not have a significant relationship with adherence [27,28,54]. Also, projects carried out with diabetic subjects coincided in stating that neither age, sex, marital status nor level of study are related to the level of adherence to treatment [55,56]. The results obtained in our research are in line with those obtained in these studies.

Finally, in relation to the duration of illness, we can affirm that our results also coincide with those of some studies carried out with patients diagnosed with diabetes mellitus, which show that adherence to treatment is higher in those subjects with a greater duration of illness [57,58]. Ramos et al. (2017) more specifically detected that there were more behaviours adherent to treatment after 10 years of illness [44]. He even stated that this increase in adherence also occurred during the first 2 years of illness. In line with the results of our work, in the investigation of Kirkman et al. (2015), it is stated that patients who were newly diagnosed with diabetes were significantly less likely to be adherent to treatment [43]. In our research, an increased probability of showing adherent behaviour was found when patients had been diagnosed for more than 10 years but not when they had been diagnosed for 2 years.

Thus, as we have presented in the introduction, these results would be useful so that, as a final objective, nursing could enhance the therapeutic management of patients. This is essential because inadequate management of the therapeutic regimen becomes a threat to the health, well-being and quality of life of the subjects [59]. 

Limitations: Different authors defend that adherence to treatment is a highly complex phenomenon, in which multiple factors intervene. Thus, there are studies that have demonstrated the relationship of other variables with adherence to treatment in chronic diseases that have not been included in our study, which is their weakness. Among these variables, the secondary effects of treatment, doctor-patient relationship, social support and comorbidities with other pathologies stand out [24,60]. On the other hand, we highlight as strengths the use of a larger sample than those used in other studies [61,62,63,64,65]. Future research should take into account other variables not considered in this study and which may be relevant but which, in order to seek parsimony in the research, we did not include.

## 5. Conclusions

Taking into account the results of the aforementioned analysis, we can affirm that the subjects with a diagnosis of HIV who use emotional coping strategies less frequently have greater adherence to treatment. Therefore, emotional coping strategies are presented as a significant variable, which allows for predicting the level of adherence to treatment. However, these data were only evidenced in subjects with HIV but not in subjects with diabetes. In parallel, only in patients with diabetes did the duration of illness have an influence on the level of adherence to treatment. Thus, it has been confirmed that diabetic subjects who have had more than 10 years of disease duration have greater adherence than patients who have had the disease 1–5 years.

However, the sociodemographic characteristics studied (sex, age, marital status and educational level) are not variables related to the level of adherence to treatment. On the other hand, rational coping strategies, or those based on the use of religion and avoidance, do not appear as significant variables that allow us to estimate adherence to treatment. These results coincide with the two subsamples studied, subjects with diabetes and subjects with HIV.

We consider it essential to know the factors related to adherence to treatment in chronic diseases. In this way, it is possible to predict and anticipate those subjects who will have poor adherence to treatment. Thus, the conclusions obtained are of great interest among nursing staff for the development of health programs that increase the patient’s skills to promote and maintain the management of the therapeutic regimen.

However, the results of this study show that the factors that predict adherence to treatment are different for each chronic disease. Therefore, it is essential to continue expanding the present study with other chronic pathologies.

## Figures and Tables

**Table 1:** Sociodemographic variables based on health status.

	HIV		Diabetes		Total		$\chi^{2}$	TE	*p*
	N	%	N	%	N	%			
N participants	199	49.8%	201	50.2%	400	100%			
Sex									
Woman	48	24.1%	58	28.9%	106	26.5%	1.151	0.054	0.283
Man	151	75.9%	143	71.1%	294	73.5%			
Civil status									
Married/couple	58	29.1%	123	61.2%	181	45.3%	42.484	0.322	0.000
Single/widowed/others	117	58.8%	64	31.8%	181	43.3%			
Separated/divorced	24	12.1%	14	7.0%	38	9.5%			
Level of studies									
Secondary or lower	168	84.4%	160	76.9%	328	82%	1.574	0.063	0.210
Superior	31	15.6%	41	20.4%	72	18%			
Age									
43 years or younger	50	25.1%	46	22.9%	96	24%	5.521	0.117	0.137
44 to 50 years	61	30.7%	63	31.3%	124	31%			
From 51 to 55 years old	57	28.6%	44	21.9%	101	25.3%			
56 years or older	31	15.6%	48	23.9%	79	19.8%			

N: Number of subjects; %: percentage, $\chi^{2}:\;\mathrm{chi}-\mathrm{square}\;$ TE: effect size; *p: p*-value.

**Table 2:** Sociodemographic variables according to adherence.

	Adherence		Non-Adherence		TOTAL		$\chi^{2}$	TE	*p*
	N	%	N	%	N	%			
N participants	264	34.0%	136	66.0%	400	100%			
Sex									
Woman	68	25.8%	38	27.9%	106	26.5%	0.220	0.023	0.639
Man	196	74.2%	98	72.1%	294	73.5%			
Civil status									
Married/couple	121	45.8%	60	44.1%	181	45.25%	1.229	0.055	0.541
Single/widowed/others	121	45.8%	60	44.1%	181	45.25%			
Separated/divorced	22	8.3%	16	11.8%	38	9.5%			
Level of studies									
Secondary or lower	215	81.4%	113	83.1%	328	82%	0.165	0.020	0.684
Superior	49	18.6%	23	16.9%	72	18%			
Age									
43 years or younger	69	26.1%	27	19.9%	96	24%	5.064	0.113	0.167
44 to 50 years	77	29.2%	47	34.6%	124	31%			
From 51 to 55 years old	61	23.1%	40	29.4%	101	25.25%			
56 years or older	57	21.6%	22	16.2%	79	19.75%			

N: Number of subjects; %: percentage, $\chi^{2}:\;\mathrm{chi}-\mathrm{square}\;$ TE: effect size; *p*: *p*-value.

**Table 3:** Variables not included in the null model. HIV subjects.

	Score	gl	*p*
Level of studies	3.804	1	0.051
Sex	0.223	1	0.637
Marital status: Married/partner	0.827	2	0.661
Marital status: Single/widowed/others	0.604	1	0.437
Marital status: Separated/divorced	0.589	1	0.443
Age: 43 years or younger	3.714	3	0.294
Age: 44 to 50 years	1.546	1	0.214
Age: 51 to 55 years	0.166	1	0.684
Age: 56 years or older	0.011	1	0.918
Duration of the disease: 1–5 years	3.612	2	0.164
Duration of the disease: 5–10 years	1.575	1	0.209
Duration of the disease: More than 10 years	3.606	1	0.058
Emotional coping strategies	7.767	1	0.005
Rational coping strategies	5.610	1	0.018
Other types of coping strategies	0.240	1	0.624

Gl: degrees of freedom; *p*: *p*-value.

**Table 4:** Variables included in the proposed model. HIV subjects.

	B	E.T.	Wald	gl	*p*	Exp(B)
Emotional coping strategies	−0.065	0.024	7.501	1	0.006	0.937
Constant	1.842	0.474	15.085	1	0.000	6.310

B: regression coefficient; E.T.: standard error; gl: degrees of freedom; *p*: *p*-value; Exp(B): exponential of the regression coefficient.

**Table 5:** Tests on the adequacy of the model. HIV sujects.

	Value	*p*
% well classified cases *	65.800%	-
Cox and Snell’s R^2^	0.038	-
Nagelkerke’s R^2^	0.053	-
Hosmer-Lemeshow	3.923	0.789

* cut point: 0.6; *p*: *p*-value.

**Table 6:** Variables not included in the null model. Diabetes Mellitus sujects.

	Score	gl	*p*
Sex	0.035	1	0.851
Marital status: Married/partner	0.701	2	0.704
Marital status: Single/widowed/others	0.032	1	0.857
Marital status: Separated/divorced	0.592	1	0.442
Age: 43 years or younger	4.075	3	0.253
Age: 44 to 50 years	0.083	1	0.773
Age: 51 to 55 years	2.374	1	0.123
Age: 56 years or older	2.811	1	0.094
Duration of the disease: 1–5 years	6.817	2	0.033
Duration of the disease: 5–10 years	0.165	1	0.685
Duration of the disease: More than 10 years	6.185	1	0.013
Emotional coping strategies	0.002	1	0.964
Rational coping strategies	0.165	1	0.684
Other types of coping strategies	1.925	1	0.165

gl.: degrees of freedom grados de libertad; *p*: *p*-value.

**Table 7:** Variables included in the proposed model. Diabetes Mellitus sujets.

	B	E.T.	Wald	gl	*p*	Exp(B)
Duration of the disease: 1–5 years			6.618	2	0.037	
Duration of the disease: 5–10 years	0.275	0.358	0.590	1	0.443	1.316
Duration of the disease: More than 10 years	0.974	0.381	6.546	1	0.011	2.649
Constant	0.318	0.232	1.879	1	0.170	1.375

B: regression coefficient; E.T.: standard error; gl: degrees of freedom; *p*: *p*-value; Exp(B): exponential of the regression coefficient.

**Table 8:** Tests on the adequacy of the model. Shows Diabetes Mellitus.

	Valor
% well classified cases *	60.500
Cox and Snell’s R^2^	0.035
Nagelkerke’s R^2^	0.048

* cut point: 0.6; *p*: *p*-value.

## Data Availability

The data that support the findings of this study are available on request from the corresponding author. The data are not publicly available due to privacy or ethical restrictions.

## References

[1] Fernández-Lázaro C.I., García-González J.M., Adams D.P., Fernandez-Lázaro D., Mielgo-Ayuso J., Caballero-García A., Moreno F., Córdova A., Miron-Canelo J.A. (2019). Adherence to treatment and related factors among patients with chronic conditions in primary care: A cross-sectional study. BMC Fam. Pract..

[2] Dios-Guerra C., Pérula L.A. (2012). Factors related to Ineffective Handle of Therapeutic Regimen (IHTR) in chronic patients of nursing consultation. Index Enferm..

[3] Danielson E., Melin-Johansso C., Modanloo M. (2019). Adherence to treatment in patients with chronic diseases: From alertness to persistence. Int. J. Community Based Nurs. Midwifery.

[4] Iancu M.A., Mateiciuc I.I., Stanescu A.A., Matei D., Diaconu C.C. (2020). Therapeutic Compliance of Patients with Arterial Hypertension in Primary Care. Medicina.

[5] Ministerio de Sanidad Consumo y Bienestar Social (2019). Vigilancia Epidemiológica del VIH y Sida en España 2018: Sistema de Información sobre Nuevos Diagnósticos de VIH y Registro Nacional de Casos de Sida.

[6] Sax P.E. (2019). Acute and Early HIV Infection. Clinical Manifestations and Diagnosis. https://www.uptodate.com/contents/acute-and-early-hiv-infection-clinical-manifestations-and-diagnosis.

[7] Varela-Arévalo M., Hoyos-Hernández P. (2015). Adherence to treatment for HIV/AIDS: Beyond the uptake of antiretrovirals. Rev. Salud Pública.

[8] Mitzel L.D., Vanable P.A. (2019). Necessity and concerns beliefs and HIV medication adherence: A systematic review. J. Behav. Med..

[9] Bezabhe W.M., Chalmers L., Bereznicki L.R., Peterson G.M. (2016). Adherence to Antiretroviral Therapy and Virologic Failure. Medicine.

[10] Kangethe A., Polson M., Lord T.C., Evangelatos T., Oglesby A. (2019). Real-world health plan data analysis: Key trends in medication adherence and overall costs in patients with HIV. J. Manag. Care Spec. Pharm..

[11] Hines D.M., Ding Y., Wade R.L., Beaubrun A., Cohen J.P. (2019). Treatment adherence and persistence among HIV-1 patients newly starting treatment. Patient Prefer. Adherence.

[12] Sánchez-Rivero I., Madoz-Gúrpide A., Parro-Torres C., Hernández-Huerta D., Mangado E.O. (2020). Coverage and Adherence of Antiretroviral Therapy Among Chinese HIV-positive Men Who Have Sex with Men With High CD4 Counts in the Era of “Treat All”. Adicciones.

[13] Costa J.D.M., Torres T.S., Esteves L., Mendes P. (2018). Adherence to antiretroviral therapy for HIV/AIDS in Latin America and the Caribbean: Systematic review and meta-analysis. J. Int. AIDS Soc..

[14] Gutiérrez-Gabriel I., Godoy-Guinto J., Lucas-Alvarado H., Pineda-Germán B., Vázquez-Cruz E., Hernández-De a Rosa M.C., Sosa-Jurado F. (2019). Quality of life and psychological variables affecting adherence to antiretroviral treatment in Mexican patients with HIV/AIDS. Rev. Chil. Infectología.

[15] López M.J., Docampo M. (2018). Change over time in prevalence of diabetes mellitus (DM) in Spain (1999–2014). Endocrinol. Diabetes Nutr..

[16] Ramirez S., Villa-Ruano N., García D. (2017). Genetic epidemiology on causal theories and pathogenesis of type 2 diabetes mellitus. Gac. Médica México.

[17] Saeedi P., Petersohn I., Salpea P., Malanda B., Karuranga S., Unwin N., Colagiuri S., Guariguata L., Motala A., Ogurtsova K. (2019). Global and regional diabetes prevalence estimates for 2019 and projections for 2030 and 2045: Results from the International Diabetes Federation Diabetes Atlas, 9 th edition. Diabetes Res. Clin. Pract..

[18] Orozco-Beltrán D., Mata-Cases M., Artola S., Conthe P., Mediavilla J., Miranda C. (2016). Adherence of Type 2 Diabetes Mellitus approach: Current situation and possible solutions. Aten. Primaria.

[19] Ting C.Y., Ahmad Zaidi Adruce S., Lim C.J., Abd Jabar A.H.A., Ting R.S.K., Ting H., Ting H., Osman N.A., Ngau E., Talin B.A. (2020). Effectiveness of a pharmacist-led structured group-based intervention in improving medication adherence and glycaemic control among type 2 diabetes mellitus patients: A randomized controlled trial. Res. Soc. Adm. Pharm..

[20] Shrivastava S., Shrivastava P., Ramasamy J. (2013). Role of self-care in management of diabetes mellitus. J. Diabetes Metab. Disord..

[21] Krass I., Schieback P., Dhippayom T. (2015). Systematic Review or Meta-analysis. Adherence to diabetes medication: A systematic review. Diabet. Med..

[22] Iniesta-Sánchez J., Abad-Corpa E., Royo-Morales T., Sáez A., Rodriguez J.J., Carrillo A. (2016). Impact assessment of a plan of nursing care of patients diagnosed with COPD nurse “Ineffective management of therapeutic regimen,” in terms of improving the nursing outcomes (NOC) “Knowledge of therapeutic regime”. Enferm. Glob..

[23] Blanco J.A., Hernández S., Botas P., Rodríguez-Rodero S., Morales P., Naya L.D., Menéndez-Torre E., Delgado E. (2019). Gender differences in the mortality of people with type 2 diabetes: Asturias Study 2018. Gac. Sanit..

[24] Menditto E., Cahir C., Aza-Pascual M., Bruzzese D., Poblador-Plou B., Malo S., Costa E., González-Rubio F., Gimeno-Miguel A., Orlando V. (2018). Adherence to chronic medication in older populations: Application of a common protocol among three European cohorts. Patient Prefer. Adherence.

[25] Corallo P.S.Y., Bonanno M.C.S., Marcella D.C., Carmela R., Edoard S., GianGaetano D.A., Viviana L.B., Giuseppo V., Plácido B., Silvia M. (2019). Therapeutic adherence and coping strategies in patients with multiple sclerosis. Medicine.

[26] Hwang H.C., Kim H.R., Han D.H., Hong J.S., Jeong S., Shin J., Kim S.M., Hwang J.H., Kim S. (2018). Influence of Major Coping Strategies on Treatment Non-adherence and Severity of Comorbid Conditions in Hemodialysis Patients. J. Korean Med. Sci..

[27] Abadiga M., Hasen T., Mosisa G., Abdisa E. (2020). Adherence to antiretroviral therapy and associated factors among Human immunodeficiency virus positive patients accessing treatment at Nekemte referral. PLoS ONE.

[28] Orellana-Zanabria G., Morales-Rezza E. (2019). Factors associated with adherence to HAART in patients with HIV/AIDS in the central hospital of the police forces. An. Fac. Med..

[29] Sabino T.E., Avelino-Silva V.I., Cavalcantte C., Goulart S.P., Luiz O.C., Fonseca L.A.M., Casseb J.S. (2021). Adherence to antiretroviral treatment and quality of life among transgender women living with HIV/AIDS in São Paulo, Brazil. AIDS Care.

[30] Morisky D.E., Green L.W., Levine D.M. (1986). Concurrent and predictive validity of a self-reported measure of medication adherence. Med. Care.

[31] Sandín y Chorot B. (2002). The Coping Strategies Questionnaire: Development and preliminary validation. Rev. Piscopatoogia Piscologia Clin..

[32] Khechane N., Mwaba K. (2004). Treatment adherence and doping with stress among black South African hemodialysis patients. Soc. Behav. Personal..

[33] Sampaio de Brito D.C., Oliveira E., Dos Santos F.R., Basile Colugnati F., Lucchetti G., Sanders-Pinheiro H. (2016). Stress, coping and adherence to immunosuppressive medications in kidney transplantation: A comparative study. Sao Paulo Med. J..

[34] Weaver K.E., Llabre M., Durán R.E., Antoni M.H., Ironson G., Penedo F.J., Schneiderman N.A. (2005). Stress and Coping Model of Medication Adherence and Viral Load in HIV-Positive Men and Women on Highly Active Antiretroviral Therapy (HAART). Health Psychol..

[35] Delmas P., Delpierre C., Côté J., Lauwers-Cancès V., Delon S. (2008). Study of the Promosud Cohort. Predictors of Adherence to Treatment Plans by French Patients Living With HIV. Perspect. Enfermo.

[36] Ahumada M., Escalante E., Santiago I. (2011). Preliminary study of relations between coping strategies, social support and adherence to treatment in people living with HIV/AIDS. Subj. Procesos Cogn..

[37] Lyimo R.A., Stutterheim S.E., Hospers H.J., Glee T. (2014). Stigma, Disclosure, Coping, and Medication Adherence Among People Living with HIV/AIDS in Northern Tanzania 1. AIDS Patient Care STDS.

[38] Malow R., Devieux J., Stein J., Rosenberg R., Jean-Gilles M., Attonito J., Koenig S.P., Raviola G., Sévère P., Pape J. (2013). Depression, Substance Abuse and Other Contextual Predictors of Adherence to Antiretroviral Therapy (ART) Among Haitians. AIDS Behav..

[39] Pedraza G.L., Vega C.Z. (2018). Stress, coping, emotions and therapeutic adherence in diabetic patients. Eureka.

[40] Ortiz M. (2006). Stress, Copying Style, and Treatment Adherence in Adolescents Sufferingfrom Type I Diabetes. Ter. Psicol..

[41] Ortiz M., Ortiz E., Gómez D. (2011). Psychosocial Factors Associated with Adherence to Type 2 Diabetes Mellitus Treatment. Ter. Psicológica.

[42] Smalls B.L., Walker R.J., Hernandez-Tejada M.A., Campbell J.A., Davis K.S., Egede L.E. (2012). Associations between coping, diabetes knowledge, medication adherence and self-care behaviors in adults with type 2 diabetes. Gen. Hosp. Psychiatry.

[43] Kirkman M.S., Rowan M.T., Levin R., Fonseca V.A., Schmittdiel J.A., Herman W.H., Aubert R.E. (2015). Determinants of Adherence to Diabetes Medications: Findings from a Large Pharmacy Claims Database. Diabetes Care.

[44] Ramos Y., Morejón R., Gómez M., Reina M.E., Rangel C., Cabrera Y. (2017). Therapeutic adherence in patients with type 2 diabetes mellitus. Rev. Final..

[45] Canales S., Barra E. (2014). Self-efficacy, social support and adherence to treatment in adult patients with type 2 diabetes. Psicol. Salud.

[46] Domínguez E. (2013). Social inequalities and diabetes mellitus. Rev. Cuba. Endocrinol..

[47] Fornos-Pérez J.A., Andrés-Rodríguez N.F., Andrés-Iglesias J.C., Mera-Gallego R., Mera-Gallego I., Penín-Álvarez Ó., Brizuela-Rodicio L. (2017). Assessment of compliance with hypoglycemic and antihypertensive treatments in Galicia. Farm. Comuniarios.

[48] Martín L.A., Bayarre V.H., Corugedo R.M.C., Vento I.F., La Rosa M.Y., Orbay A.M.C. (2015). Adherence to treatment observed in hypertensive patients from health areas of three cuban provinces. Rev. Cuba. Salud Publica.

[49] Quiñones Á., Ugarte C., Chávez C., Mañalich J. (2018). Psychological variables associated with adherence to treatment and complications in patients with type 2 diabetes mellitus. Rev. Med. Chile.

[50] Camargo C., Cavassan N., Tasca K., Meneguin S., Miot H., Souza L. (2019). Depression and coping are associated with failure of adherence to antiretroviral therapy among people living with HIV/AIDS. Res. Hum. Retrovir..

[51] Zhao H., Zhang N., Ho V., Ding M., He W., Niu J., Yang M., Du X.L., Zorzi D., ChavezMacGregor M. (2018). Adherence to treatment guidelines and survival for older patients with stage II o III colon cancer in Texas from 2001 to 2011. Cancer.

[52] Camp Y., Vrijens B., Elseviers M.M. (2014). Adherence to phosphate binders in hemodialysis patients: Prevalence and determinants. J. Nephrol..

[53] Ossareh S., Tabrizian S., Zebarjadi M., Joodat R. (2014). Prevalence of Depression in Maintenance Hemodialysis Patients with Adherence to Medications. Iran J. Kidney Dis..

[54] Lemos L.D.A., Teles M.L., Reis R.K., Ferrer A.C., Gir E., Gimeniz M. (2016). Adherence to antiretrovirals in people coinfected with the human immunodeficiency virus and tuberculosis. Rev. Lat. Am. Enferm..

[55] Pereira A., De Sousa B., Borges D.S., García J., Silva F.V., Coelho M.M., Júnior R. (2015). Adherence to the treatment with oral antidiabetic medications in primary health care. Rev. Rene.

[56] Leites-Docío A., García-Rodríguez P., Fernández-Cordeiro M., Tenorio-Salgueiro L., Fornos-Pérez J.A., Andrés-Rodríguez N.F. (2019). Evaluation of non-adherence to hipoglucemiary treatment in community pharmacy. Farm. Comuniarios.

[57] Klinovszky A., Kiss I.M., Papp-Zipernovszky O., Lengyel C., Buzás N. (2019). Associations of different adherences in patients with type 2 diabetes mellitus. Patient Prefer. Adherence.

[58] Marinho F.S., Moram C.B.M., Rodrigues P.C., Leite N.C., Salles G.F., Cardoso C.R.L. (2018). Treatment Adherence and Its Associated Factors in Patients with Type 2 Diabetes: Results from the Rio de Janeiro Type 2 Diabetes Cohort Study. J. Diabetes Res..

[59] Bakker R.H., Kastermans M.C., Dassen T.W. (1995). An analysis of the nursing diagnosis ineffective management of therapeutic regimen compared to noncompliance and Orem’s self-care deficit theory of nursing. Nurs. Diagn..

[60] Escandón-Nagel N., Azócar B., Pérez C., Matus V. (2015). Adherence to treatment in type 2 diabetes and itsassociation with quality of life and depression. Rev. Psicoter..

[61] Ceylan E., Koç A., Inkaya A.Ç., Ünal S. (2019). Determination of medication adherence and related factors among people living with HIV/AIDS in a Turkish university hospital. Turk. J. Med. Sci..

[62] Chirambo L., Valeta M., Banda T.M., Nyondo-Mipando A.L. (2019). Factors influencing adherence to antiretroviral treatment among adults accessing care from private health facilities in Malawi. BMC Public Health.

[63] Ghanbari A., Khiaban M.O., Aslani A., Faraji A.R., Mohammadi M., Kazemi A.F. (2019). Assessment of adherence to therapy and exploring of barriers and facilitators in HIV positive patients in Tabriz-Iran: A mixed method study protocol. Reprod. Health.

[64] Hushie M. (2019). Exploring the barriers and facilitators of dietary self-care for type 2 diabetes: A qualitative study in Ghana. Health Promot. Perspect..

[65] Radwan M., Sari A.A., Rashidian A., Takian A., Elsous A., Abou-Dagga S. (2018). Factors hindering the adherence to clinical practice guideline for diabetes mellitus in the Palestinian primary healthcare clinics: A qualitative study. BMJ Open.

