# Correction: Rivera-Picón et al. Factors Associated with Adherence to Treatment in Patients with HIV and Diabetes Mellitus. *J. Pers. Med.* 2023, *13*, 269

**DOI:** 10.3390/jpm13081215

**Published:** 2023-07-31

**Authors:** Cristina Rivera-Picón, María Hinojal Benavente-Cuesta, María Paz Quevedo-Aguado, Juan Luis Sánchez-González, Pedro Manuel Rodríguez-Muñoz

**Affiliations:** 1 Faculty of Health Sciences, Nursing, Pontifical University of Salamanca, 37002 Salamanca, Spain; criverapi@upsa.es (C.R.-P.); mhbenaventecu@upsa.es (M.H.B.-C.); mpquevedoag@upsa.es (M.P.Q.-A.); 2 Faculty of Nursing and Physiotherapy, University of Salamanca, 37008 Salamanca, Spain; pedromrodriguez@usal.es; 3 Department of Nursing, Instituto Maimónides de Investigación Biomédica de Córboda, 14005 Córdoba, Spain

## Text Correction

There was an error in the original publication [1] “Factors Associated with Adherence to Treatment in Patients with HIV and Diabetes Mellitus”.

The Morisky, Green, and Levine scale (MGL) was used in the study, not the MMAS-4 as initially indicated. The term “4-item Morisky medication adherence scale” on Abstract and Section 2.3.2 needs to be replaced with “Morisky, Green, and Levine Medication Adherence Scale”.

A correction has been made to the Abstract and Section 2.3.2.

**Abstract:** We aim to identify the factors that influence the therapeutic adherence of subjects with chronic disease. The design followed in this work was empirical, not experimental, and cross-sectional with a correlational objective. The sample consisted of a total of 400 subjects (199 patients with HIV and 201 patients with diabetes mellitus). The instruments applied for data collection were a sociodemographic data questionnaire; the Morisky, Green, and Levine Medication Adherence Scale (MGL); and the Coping Strategies Questionnaire. In the group of subjects with HIV, the use of emotional coping strategies was related to lower adherence to treatment. On the other hand, in the group of subjects with diabetes mellitus, the variable related to compliance with treatment was the duration of illness. Therefore, the predictive factors of adherence to treatment were different in each chronic pathology. In the group of subjects with diabetes mellitus, this variable was related to the duration of the disease. In the group of subjects with HIV, the type of coping strategy used predicted adherence to treatment. From these results, it is possible to develop health programs to promote issues ranging from nursing consultations to the adherence and treatment of patients with HIV and diabetes mellitus.

2.3.2. Morisky, Green, and Levine Medication Adherence Scale (MGL)

This questionnaire was originally developed by Morisky et al. (1986) under the name the “Morisky, Green, and Levine Medication Adherence Scale (MGL)”. The Spanish version of this questionnaire was validated by Val-Jiménez et al. (1992). The scale is made up of four questions with a dichotomous response format (Yes/No), reflecting the patient’s behavior regarding compliance. A patient shows good adherence to treatment when he/she correctly answers the four questions (No/Yes/No/No) and presents poor adherence if he/she answers three or fewer questions adequately. Therefore, through this questionnaire, patients are categorized as adherent to treatment or nonadherent to treatment [30].

Originally, the questionnaire had adequate psychometric properties with high discrimination (*ρ_bp_* = 0.43), high sensitivity (sensitivity = 0.81), and low specificity (specificity = 0.44). In Spain, this test was validated by Val-Jiménez et al. (1992) using a sample of hypertensive patients. The sensitivity was somewhat lower, while the specificity value was practically the same (sensitivity = 0.52, specificity = 0.44).

The authors state that the scientific conclusions are unaffected. This correction was approved by the Academic Editor. The original publication has also been updated.
